# The Conceptualization of Value in the Value Proposition of New Health Technologies: Comment on "Providing Value to New Health Technology: The Early Contribution of Entrepreneurs, Investors, and Regulatory Agencies"

**DOI:** 10.15171/ijhpm.2017.75

**Published:** 2017-07-03

**Authors:** Sandra C. Buttigieg, Joost van Hoof

**Affiliations:** ^1^Faculty of Health Sciences, University of Malta, Msida, Malta.; ^2^School of Social Policy, College of Social Sciences, University of Birmingham, Birmingham, UK.; ^3^Institute of Allied Health Professions, Fontys University of Applied Sciences, Eindhoven, The Netherlands.

**Keywords:** Conceptualisation of Value, New Technology, Innovations, Stakeholder Analysis

## Abstract

Lehoux et al provide a highly valid contribution in conceptualizing value in value propositions for new health technologies and developing an analytic framework that illustrates the interplay between health innovation supply-side logic (the logic of emergence) and demand-side logic (embedding in the healthcare system). This commentary brings forth several considerations on this article. First, a detailed stakeholder analysis provides the necessary premonition of potential hurdles in the development, implementation and dissemination of a new technology. This can be achieved by categorizing potential stakeholder groups on the basis of the potential impact of future technology. Secondly, the conceptualization of value in value propositions of new technologies should not only embrace business/economic and clinical values but also ethical, professional and cultural values, as well as factoring in the notion of usability and acceptance of new technology. As a final note, the commentary emphasises the point that technology should facilitate delivery of care without negatively affecting doctor-patient communications, physical examination skills, and development of clinical knowledge.

## Introduction

Lehoux et al provide a highly valid contribution in conceptualizing value in value propositions for new health technologies and developing an analytic framework that illustrates the interplay between health innovation supply-side logic (the logic of emergence) and demand-side logic (embedding in the healthcare system).^1^ The authors emphasize the importance of meaningful contributions by three key stakeholders, namely entrepreneurs, investors and regulatory agencies in the early stages of the innovation lifecycles claiming that this will more likely result in successful uptake by users and an acceptably good return on investment for investors. The authors also highlight, through the literature, qualitative research and discussion, the multifactorial complexities and challenges involved in balancing the innovation policy, namely the supply side logics as determined by financial markets and business media, and the health policy demand-side logics that include health technology assessment, reimbursement and procurement. The authors however acknowledge, “New technologies constitute an important cost-driver in healthcare, but the dynamics that lead to their emergence remains poorly understood…” (p. 1).

In this commentary, we would like to put forward several points, as part of the commentary of this article.

## Stakeholder Analysis

Lehoux et aldecided to focus on three key stakeholders in their study. Although entrepreneurs involve clinical teams to provide valuable information regarding patient needs and health benefits of new technologies, capital investors look out for business opportunities of innovations to curb down health risks of extant technologies or clinical practice, and regulatory agencies aim towards achieving patient safety, and the efficacy and quality of technology, the minimally consulted users/patients/clients often have to take what is on offer without a choice for alternatives. In addition, the client system revolving around the patient, in particular the informal caregiver is often overlooked. In the delivery of healthcare services and technologies, the patient should be the focus of attention. Van Hoof et al,^2^ Holtkamp et al,^3^ and van Hoof & Verkerk^4^ have come up with various frameworks for the incorporation of technology philosophy in order to help identify and map the needs of patients (and other stakeholders) within different user contexts. Such frameworks, however, do not deny the complexity of the intertwined world of stakeholder needs. In particular, for technologies that are still to be made available, defining all the potential stakeholders is challenging. Moreover, in terms of user acceptance, consulting potential end-users during the pre-implementation phase of new technologies can lead to skewed representations towards the usefulness of such devices that would have been more mature and crystallised if people had the chance to experience and engage with the devices in question.^5^ Therefore, it is important to envisage the potential impact of future technology, and this entails primarily categorizing potential stakeholder groups that could be affected, as well as recognising the differences between pre- and post-implementation research.^6^

A suggested approach is using temporal stakeholder and event analysis, which transfers learning from different stakeholder groups’ experiences with previous technologies, to new technologies or systems. This analysis provides a structure that captures the lifecycle of technologies and outlines the impact on different stakeholder groups at each stage of the lifecycle.^6^ A robust stakeholder analysis provides the necessary premonition of potential hurdles in the development, implementation and dissemination of a new technology. A comprehensive analysis will more likely guide the course of development of technology early on, while considering affected stakeholders and the wider community.^7^

On the other hand, the identification of too many stakeholders may be problematic in that these have specific interests that may separately or collectively impede the implementation of new technology, regardless of the clients’ intentions.^2,8^ On many occasions, the client’s consent is taken for granted. The example of the home telehealth solution reveals that the chronically ill and older adults, potentially identified at the end of the process, are accidental stakeholders.^8^

## Defining “Value” in Value Propositions of New Technologies

The manner in which the three key stakeholders mentioned in the study define value is a clear reflection of the often-limited business/economic focus adopted. The examples of innovations in the article alert us of this limitation and invite us to consider a broader and more comprehensive conceptualization of value in value propositions of new technologies. Additionally, the qualitative study highlights the vested interests of the three stakeholders, and therefore the fragmented manner in which value is conceptualized. For example, the authors lament, “Investors support technologies that generate health gains by accident, not by design.”^1^ The article also refers to investors, who because of their desire for a quick return on investment, and their aim to bring the venture to the most profitable exit, will only provide support for a limited period of time despite evidence of patient benefits and value creation for health systems.

Entrepreneurs, who tap on their healthcare experience, seem to have a more balanced consideration of not only having the proposed technology generating revenues but also a capacity to generate health benefits and therefore do have a patient focus. The regulatory agencies also have a somewhat specific remit, namely that of focusing on safety and efficacy of new technologies relying on science, expertise and judgment when executing appraisals. While the values defined are of critical consideration, we propose stakeholders to collectively consider ethical concepts apart from the business/economic and clinical values that are described in more detail below.

The first that comes to mind are the ethical values and aspects when proposing and considering new technologies.^9^ Technology is considered to influence various human experiences, amongst them health.^10^ Although it influences social changes, and could be a source of power, vulnerability, and inequality, new technologies are still too often introduced with little critical reflection on their impacts.^10^ This is of particular concern in healthcare when the problematic allocation of scarce resources should leave little room for experimentation in view of the ever-rising healthcare costs and health inequalities.

The second concept is the consideration of professional values of healthcare professionals, whose main goal is their ability to satisfy the needs of their patients and clients.^11^ In particular, professionals delivering chronic care value inter-collegial respect. Additionally, clinicians do not relate technological solutions for clinical problems. Therefore, for success in the implementation of new technologies, it is essential that these values are not disrupted and that clinicians should receive solid support and education to effectively link technological solutions to care recipients’ needs.^11^

The third concept is the consideration of the social and cultural values of the value proposition. The extent to which a technology is effective in the world does not only depend on its technical aspects but also on how successful it is to match the social and ecological contexts where it is implemented.^12^ See, for instance, Moors and Peine,^13^ who discussed how to value health innovations within a broader socio-cultural context. Therefore, stakeholders should also consider the social and cultural aspects of technology in its design, implementation and dissemination.^12^

The fourth concept is the consideration of usability, ie, user-oriented approach, which is considered an important determinant of acceptance.^8^ If a new technology satisfies three properties, namely effectiveness, efficiency and satisfaction, then it is more likely to be high on usability. Additionally, memorability and learnability are also considered important.^8^

New technologies, if introduced successfully, will always to some extent disrupt existing ethical, professional or cultural practices. This encompasses more than just meeting user needs or professional values. There will always be socio-material practices that arise during the implementation, which are the “locale” in which old values are contested and new values arise. These emerging and evolving values are critical in assessing a technology and determining its “success.” The distinction between a prosthetic logic (in which technologies are assessed according to how well they support the model of existing values) and a habilitating logic (in which technologies are assessed according to how far they allow new values to emerge reflexively) might help in this regard.^13^

## A Note on Health Technology Assessment

The focus of technological assessments tends to be mostly on the positive and negative impacts or effects of a technology without much consideration of the manner with which they restructure our physical and social worlds.^14^ Winner argues, “The capacity and willingness to reflect on the significance of technology and to critically evaluate new technologies lags far behind our capacity for creating and disseminating technologies” (p. 48).^14^ Unless a more robust assessment is used that encompasses the biopsychosocial model of care, new technologies pose numerous challenges in the various stages from the development to adoption and subsequent sustainability of the uptake.

## Conclusion

In this commentary, we have highlighted only some aspects of how new health technologies can provide greater value to end-users and to society as a whole. The subject area is complex, challenging and continues to confound leaders of health systems. Our commentary is intended to shed more light on what Lehoux et al masterfully provided in their study. As a final note, we believe that technology should facilitate the delivery of care without negatively affecting doctor-patient communications, physical examination skills, the perception of safety and security among patients, and the development of clinical knowledge.^15^ A potential side effect of the increased usage of technology in healthcare is the rise in costs that may hamper the overall accessibility of cure and care services, both from a patient and a professional perspective. Indeed, since clinicians are becoming more dependent on technology, in particular for accessing patient information, reaching diagnoses and delivering care, undergraduate and professional development training on using medical technology in the domains of cure and care has become a must to ensure autonomy while interfacing with medical technology.^15^

## Ethical issues

Not applicable.

## Competing interests

Authors declare that they have no competing interests.

## Authors’ contributions

SCB and JvH have discussed the outline for the paper. SCB has drafted the paper, which JvH has critically read and amended. Both SCB and JvH have revised after receiving reviewers’ feedback.

## Authors’ affiliations

^1^Faculty of Health Sciences, University of Malta, Msida, Malta. ^2^School of Social Policy, College of Social Sciences, University of Birmingham, Birmingham, UK. ^3^Institute of Allied Health Professions, Fontys University of Applied Sciences, Eindhoven, The Netherlands.
